# The role of SIRT2 in vascular‐related and heart‐related diseases: A review

**DOI:** 10.1111/jcmm.16618

**Published:** 2021-05-24

**Authors:** Boquan Wu, Shilong You, Hao Qian, Shaojun Wu, Saien Lu, Ying Zhang, Yingxian Sun, Naijin Zhang

**Affiliations:** ^1^ Department of Cardiology The First Hospital of China Medical University Shenyang China

**Keywords:** diabetic cardiomyopathy, heart failure, ischaemia‐reperfusion injury, oxidative stress, pathological cardiac hypertrophy, SIRT2

## Abstract

At present, cardiovascular disease is one of the important factors of human death, and there are many kinds of proteins involved. Sirtuins family proteins are involved in various physiological and pathological activities of the human body. Among them, there are more and more studies on the relationship between sirtuin2 (SIRT2) protein and cardiovascular diseases. SIRT2 can effectively inhibit pathological cardiac hypertrophy. The effect of SIRT2 on ischaemia‐reperfusion injury has different effects under different conditions. SIRT2 can reduce the level of reactive oxygen species (ROS), which may help to reduce the severity of diabetic cardiomyopathy. SIRT2 can affect a variety of cardiovascular diseases, energy metabolism and the ageing of cardiomyocytes, thereby affecting heart failure. SIRT2 also plays an important role in vascular disease. For endothelial cell damage used by oxidative stress, the role of SIRT2 is bidirectional, which is related to the degree of oxidative stress stimulation. When the degree of stimulation is small, SIRT2 plays a protective role, and when the degree of stimulation increases to a certain level, SIRT2 plays a negative role. In addition, SIRT2 is also involved in the remodelling of blood vessels and the repair of skin damage.

## 1 INTRODUCTION

Cardiovascular diseases have become one of the important factors that endanger human health, and post‐translational modifications of related proteins play an important role in this.^1^, ^2^, ^3^, ^4^, ^5^ Among them, acetylation and deacetylation have increasingly become the focus of research. The seven‐member sirtuins family (SIRT1‐7) belongs to the third deacetylase. In order to catalyse the deacetylation reaction, this protein requires the presence of nicotinamide adenine dinucleotide (NAD^+^) to have deacetylation activity. Sirtuins are involved in a variety of physiological and pathological activities in the human body, including ageing, energy responses to low calorie availability and stress resistance, apoptosis, inflammation, regulate mitochondrial biogenesis and biological rhythm. We currently know the substrates of the sirtuins family proteins including p53,^6^ nuclear factor‐kappa B (NF‐κB),^7^ forkhead box protein O1 (FOXO1),^8^ forkhead box protein O3 (FOXO3),^9^ peroxisome proliferator‐activated receptor‐γ (PPAR‐γ),^10^ peroxisome proliferator‐activated receptor gamma coactivator 1‐alpha (PGC1‐α),^11^ acetyl‐CoA synthetases (AceCSs),^12^ α‐tubulin,^13^ cell division cycle 20 (CDC20),^14^ CDC20‐homologue 1 (CDH1),^14^ phosphoenolpyruvate carboxykinase (PEPCK1),^15^ histone H3 lysine 56 (H3K56)^16^ and histone H4 lysine 16 (H4K16).^17^ The positioning of sirtuins in the cells is also different. At the same time, the substrates and binding partners used are also different.^18^ For SIRT2, it is mainly located in the cytoplasm, but under certain conditions, it will also enter the nucleus and play the corresponding function.^13^ At present, sirtuin 1 (SIRT1) is the most widely studied in the cardiovascular system, and SIRT2 is relatively little studied. In this review, we summarized the different functions and simple mechanisms of SIRT2 in the cardiovascular system, which is expected to contribute to finding therapeutic targets for cardiovascular diseases.

## 2 SIRT2 AND HEART‐RELATED DISEASES

### 2.1 SIRT2 and pathological myocardial hypertrophy

Myocardial hypertrophy is a powerful form of compensation, which is divided into physiological myocardial hypertrophy and pathological myocardial hypertrophy. The significance of physiological myocardial hypertrophy is positive. It is made by the body to some changes in the body or some external stimuli. The adaptive changes often occur in pregnant women and athletes.^19^ This is a reversible change. After the stimulation disappears, the myocardium returns to normal levels. However, under the action of some pathological stimulation, myocardial cells will undergo pathological myocardial hypertrophy.^20^ This change is within a certain range. If the cause is persistent and cannot be eliminated, the hypertrophic cardiac muscle cannot maintain normal function for a long time and eventually turn to heart failure.^21^ Chronic heart failure generally develops gradually on the basis of compensatory hypertrophy of the myocardium.

Post‐translational modification is very important in the development of pathological myocardial hypertrophy, and acetylation is one of them.^22^ The family of histone deacetylases (HDAC) plays a key role in regulating pathological cardiac hypertrophy. HDACs are divided into four categories. Class I HDAC can promote the occurrence and development of pathological cardiac hypertrophy, which in turn aggravates the condition.^23^ Class II HDAC is contrary to it and plays a negative role in regulating cardiac hypertrophy.^24^ The sirtuins family belong to class III HDAC.^25^ And so far, the role of class IV HDAC is unclear.

There was evidence that SIRT2 is related to pathological myocardial hypertrophy and plays a negative role in regulating the occurrence and development of pathological myocardial hypertrophy, thereby reducing the severity of illness and heart failure. SIRT2 can deacetylate the liver kinase B1 (LKB1), thereby promoting the activation of the AMP‐activated protein kinase (AMPK) pathway.^26^ The loss of SIRT2 inhibits the activation of AMPK, promotes ageing‐related and/or angiotensin II (Ang II)‐induced pathological cardiac hypertrophy and weakens the metformin‐mediated cardiac protection. SIRT2 can affect the stability of microtubules (MTs) by deacetylating tubulin^27^ and has a certain effect on the FOXO3a pathway related to oxidative stress.^9^ In addition, the deacetylation of FOXO1 and PGC1‐α under the influence of SIRT2 is also related to pathological myocardial hypertrophy.^28^ At the same time, we noticed that the above four proteins, namely tubulin, FOXO3a, FOXO1, PGC1‐α are all downstream proteins of the LKB1‐AMPK signalling pathway. Therefore, it is reasonable to believe that in the protective effect of SIRT2 on pathological cardiac hypertrophy, LKB1‐AMPK signalling pathway plays a vital role. In addition to the LKB1‐AMPK pathway, the calcineurin/nuclear factor of activated T‐cells (NFAT) pathway may also be involved. NFAT is a transcription factor and NFATc2 is one of its isomers. SIRT2 can bind to NFATc2 and deacetylate it, inhibit NFATc2’s activity and then inhibit this signal transduction pathway and inhibit the occurrence of pathological myocardial hypertrophy.^29^ The latest research showed that constitutive photomorphogenic 9 signalosome complex subunit 6 (CSN6) and plant homeodomain finger protein 19 (PHF19) exacerbate the pathological myocardial hypertrophy induced by Ang II by inhibiting the expression of SIRT2, and the overexpression of SIRT2 can reduce the effect of CSN6 in promoting myocardial hypertrophy.^30^, ^31^

In short, for pathological myocardial hypertrophy, the role of SIRT2 is positive. Therefore, finding a highly effective SIRT2 agonist can effectively suppress the occurrence and development of pathological myocardial hypertrophy and then avoid the development of heart failure.

### 2.2 SIRT2 and myocardial ischaemia‐reperfusion injury

The meaning of myocardial ischaemia‐reperfusion is that the coronary artery is partially or completely acutely blocked due to some reasons, and then recanalized after a period of time. Although the perfusion of the ischaemic myocardium can be restored to normal at this time, myocardial tissue damage is instead progressive aggravation.^32^ After vascular recanalization, a series of traumatic changes in myocardial ultrastructure, energy metabolism, cardiac function and electrophysiology caused by myocardial ischaemia become more serious. At this time, the patient may experience severe arrhythmia or even sudden death.

Cardiac ischaemia or hypoxia caused by acute myocardial infarction (AMI) can cause the ATP content in cardiomyocytes to drop rapidly. In order to adapt to the hypoxic environment, cardiomyocytes obtain energy through anaerobic respiration, that is, glycolysis, which leads to accumulation of lactic acid, an increase in intracellular H+ content and activation of Na+‐H+ ion exchange channel. The energy reduction caused by hypoxia will inhibit the function of 3Na+‐2K+ ATPase and increase the accumulation of Na+in the cell. In order to expel a large amount of Na+in the cell, the 2Na+‐Ca2+ ion exchange channel is activated. Eventually, it causes Ca2+ overload in myocardial cells, triggering a series of adverse reactions.^33^ Prolonged ischaemia also induces mitochondria to release cytochrome c, which is an important protein in the classical apoptosis pathway. A prospective observational study found that the higher the level of SIRT2 in the plasma of patients with AMI, the higher the Killip class of the patient, and the lower the left ventricular ejection fraction (LVEF). This indicates that the level of SIRT2 is closely related to AMI, and AMI can be used to predict major adverse cardiovascular events (MACE) after AMI.^34^ Another study conducted on AMI patients also found that mutations in the SIRT2 gene promoter are closely related to AMI.^35^

Reperfusion can provide oxygen and nutrition, meanwhile remove toxic substances produced by necrotic cells to restore the ischaemic and hypoxic state of myocardial cells. However, the rapid recovery of oxygen content and extracellular pH^36^ will cause the accumulation of ROS^37^ and aggravate Ca2+ overload situation. This will lead to apoptosis and necrosis and furthermore aggravate pathological injury than before reperfusion. Among the present studies of sirtuins family, only SITR1 is involved in ischaemia‐reperfusion injury and few studies focussed on SIRT2. Some studies observed SIRT2 could aggravate ischaemia‐reperfusion injury by inhibiting the binding of 14‐3‐3 zeta and B‐cell lymphoma‐2 associated death promoter (BAD), furthermore releasing BAD into mitochondria from the cytoplasm. In mitochondria, BAD binds to B‐cell lymphoma‐2 (BCL‐2) and/or B‐cell lymphoma‐extra large (BCL‐XL) and inhibits the anti‐apoptotic functions of them to promote cell apoptosis. In experiments, the levels of 14‐3‐3 zeta and BAD are elevated in SIRT2 depleted H9C2 cells, and the interaction between these two proteins was enhanced.^38^ However, other studies have shown that toll‐like receptor 4 (TLR4) can reduce the expression and activity of SIRT2, increase the acetylation of p53, increase the level of ROS in cardiomyocytes and promote cell apoptosis. The overexpression of SIRT2 can inhibit the acetylation of p53 and thus inhibit the damage of TLR4 to cardiomyocytes.^39^ The reason why SIRT2 has these two opposite functions needs further research to discover.

### 2.3 SIRT2 and diabetic cardiomyopathy

Diabetes is a complex chronic disease, and it appears in multiple states worldwide.^40^ The causes of diabetes are diverse and eventually lead to absolute or relative lack of insulin, which leads to diabetes. Cardiovascular complication is one of the main complications of diabetes and one of the main causes of death of diabetic patients.^41^ The disease causes extensive focal necrosis of the myocardium on the basis of metabolic disorders^42^ and microvascular lesions, followed by abnormal heart function. The disease is characterized by atherosclerosis and left ventricular (LV) dysfunction not related to coronary artery disease, and this dysfunction changes from diastolic dysfunction to systolic dysfunction as the disease progresses.^43^ Eventually, the patients develop heart failure, arrhythmia, cardiogenic shock and even sudden death.

Microtubules are present in various cells. Their most important roles are to function as cytoskeletal filaments. MTs are composed of heterodimers containing α‐tubulin and β‐tubulin. The latter two can undergo various post‐translational modifications. After acetylation modification, this heterodimer will make MTs more stable.^44^ However, previous studies have found that in rats with type 1 diabetes mellitus (T1MD), when the content of MTs in cardiomyocytes is too high, that is, the state of MTs is stable, ventricular contractile function will be impaired,^45^ which in turn causes diabetic cardiomyopathy.

As we mentioned earlier, one of the substrates of SIRT2 is α‐tubulin. SIRT2 makes it deacetylated; therefore, the above processes will be inhibited, and eventually, the stability of MTs will be reduced. This can well relieve ventricular systolic dysfunction and delay the development of cardiomyopathy caused by diabetes.^46^ However, only T1DM is mentioned here. The relationship between type 2 diabetes mellitus (T2DM)‐induced cardiomyopathy and SIRT2 will be studied in the future.

At present, there are very few direct studies on the relationship between SIRT2 and diabetic cardiomyopathy. It is interesting that other literatures indicate the use of HDAC inhibitors, such as sodium butyrate,^47^ MPT0E014,^48^ RGFP966 (a kind of selective HDAC3 inhibitor),^49^ suberoylanilide hydroxamic acid (SAHA),^50^ can well control the occurrence and development of diabetic cardiomyopathy. However, because the sirtuins family belongs to the third category of HDACs, the effects of these two conditions for diabetic cardiomyopathy are completely opposite. This phenomenon may be caused by the interaction between different types and even different subspecies of HDACs. The specific reason needs to be studied in the future.

In addition, there are reports in the literature that the generation of high glucose‐induced ROS can cause diabetic cardiomyopathy. And experiments have shown that the use of diallyl trisulfide (DATS) can inhibit the generation of ROS, which in turn inhibits c‐Jun N‐terminal kinase (JNK)/NF‐κB signalling, thereby inhibiting high glucose‐induced myocardium apoptosis.^51^ SIRT2 can reduce the content of ROS, which provides another idea for the treatment of diabetic cardiomyopathy.

### 2.4 SIRT2 and heart failure

Heart failure is caused by impaired systolic and/or diastolic function of the heart. The amount of venous circulatory blood cannot be fully discharged from the heart, resulting in blood stasis in the venous system and insufficient blood can be perfused in the arterial system, resulting in impaired cardiac circulation. This disorder manifests as pulmonary congestion and vena cava congestion. Heart failure is not an independent disease, but the end stage of the development of heart disease. The consequences of almost all cardiovascular diseases without active treatment are heart failure, such as the aforementioned myocardial hypertrophy, myocardial ischaemia‐reperfusion injury and myocardial infarction,^52^ hypertension,^53^ valvular heart disease,^54^ cardiomyopathy,^55^ arrhythmia.^56^ The vast majority of heart failures begin with left heart failure, which first manifests as pulmonary congestion.

In addition to the aforementioned causes of heart failure, the energy metabolism in cardiomyocytes is also very important for the development of heart failure. Because the function of the heart is to transport blood to the whole body, it is working all the time, and the demand for energy is very high. So the energy metabolism in myocardial cells, especially the state of mitochondria, is closely related to heart failure. PGC1‐α is a cofactor for PPAR‐γ in adipocytes and can generate heat when the body temperature is low.^57^ In the heart, PGC1‐α is the main regulator of mitochondrial biogenesis and metabolism.^58^ For decades, the relationship between PGC1‐α and cardiac energy regulation has attracted more and more attention, and its mechanism has also been explored more and more clearly. The expression and function of PGC1‐α are key determinants of cardiac energy status. At present, studies have shown that the reduction of PGC1‐α expression is the main cause of heart failure due to mitochondrial damage and metabolic defects in the heart.^59^ At the same time, some studies have proved the relationship between SIRT1 and the expression level of PGC1‐α. It has been found in the myocardial tissue of heart failure that SIRT1 can deacetylate the histone H3 lysine 9 (H3K9) in the promoter of PGC1‐α, resulting in genes Inactivation, thereby interfering with the expression of PGC1‐α.^60^ Although the relationship between SIRT2 and PGC1‐α in cardiomyocytes and the role of SIRT2 in cardiac mitochondrial function and energy metabolism are currently unclear, related experiments in adipocytes have shown that reduced SIRT2 function directly inhibits the deacetylation of PGC1‐α and β‐oxidation and the expression of mitochondrial gene.^61^ Therefore, we speculate that SIRT2 will promote the deacetylation of PGC1‐α in cardiomyocytes, which will reduce the expression of PGC1‐α and eventually lead to heart failure. This result is hoped to be confirmed by experiments in the future.

In addition, the ageing of cardiomyocytes and heart failure is also closely related, and experiments have shown that the balance between the survival and death of cardiomyocytes plays a key role in heart failure.^62^ For SIRT2, the relationship with the nervous system was mainly studied before.^63^ Recently, it has been reported that budding uninhibited by benzimidazole‐related 1 (BUBR1) can delay the senescence of mouse cells and prolong the lifespan of mice, while SIRT2 can deacetylate BUBR1 and inhibit its degradation. This function of SIRT2 reverses the reduction in LV chamber dimension and ensures the normal heart function.^64^ SIRT2 can also regulate the activity of NFATc2 to protect the heart from diseases related to ageing of cardiomyocytes and inhibit heart failure.^29^

## 3 SIRT2 AND VASCULAR‐RELATED DISEASES

### 3.1 SIRT2 and atherosclerosis

Atherosclerosis is the main cause of coronary heart disease, cerebral infarction^65^ and peripheral vascular disease.^66^ If patients with atherosclerosis are not actively treated in time, the consequences are very serious and even life‐threatening. Risk factors for atherosclerosis include hypercholesterolemia,^67^ metabolic syndrome,^68^ hypertension,^69^ obesity^70^ and diabetes.^71^ The basis of atherosclerosis is lipid metabolism disorder.^72^ It is characterized by the lesion of the affected artery starting from the intima, generally the accumulation of lipids and complex carbohydrates, followed by bleeding and thrombosis, and then fibrous tissue hyperplasia and calcification, and the gradual transformation and calcification of the middle layer of the artery, and finally thickening and stiffening of the arterial wall, and narrowing of the vessel lumen.^73^ Lesions often involve large and medium muscular arteries. Once they develop enough to block the arterial lumen, the tissue or organ supplied by the artery will be ischaemic or necrotic.^74^ When the thrombus falls off for some reason, the thrombus will move to various organs of the body with the flow of blood flow, causing secondary damage to the remaining organs.

Macrophages play a very important role in the development of atherosclerosis.^75^ Macrophages have two phenotypes, namely "inflammatory" M1 phenotype and "regulatory" M2 phenotype, and these two phenotypes coexist in the lesion^76^ and switch back and forth according to the different conditions of the lesion,^77^ and exercise different functions. The roles of these two macrophage phenotypes in the occurrence and development of atherosclerosis is very complex. In short, the M1 phenotype inhibits the stability of atherosclerotic plaques and leads to more serious results, the M2 phenotype will make the plaque more stable and relieve the damage caused by atherosclerosis.^78^ Therefore, adjusting the ratio of M1 and M2 phenotypes in the plaque can regulate the development of atherosclerosis. Experiments have shown that overexpression of SIRT2 protein in HUVEC can reduce the expression level of inducible nitric oxide synthase (iNOS) (macrophage M1 type marker) and increase the expression level of arginase‐1 (ARG‐1) (macrophage M2 type marker). This proved that SIRT2 can transform macrophages in HUVEC from M1 type to M2 type, thereby stabilizing plaque in atherosclerosis. SIRT2 plays a protective role in this.^79^

Under normal circumstances, vascular endothelial cells will produce physiological doses of ROS to participate in the transmission of cell signals, but excessive ROS will lead to the generation of oxidative stress, which in turn causes atherosclerosis.^80^ In atherosclerotic HUVEC, if MicroRNA‐140‐5p is overexpressed, the content of ROS and malondialdehyde (MDA) in the cell increases, while the expression of SIRT2 is inhibited. Moreover, SIRT2 agonists can also inhibit the positive effect of MicroRNA‐140‐5p on oxidative stress. Therefore, it is reasonable to believe that SIRT2 mediates the effect of MicroRNA‐140‐5p on oxidative stress, and the function of SIRT2 is to suppress negative effects of oxidative stress.^81^ In addition, in the experiment of tumour necrosis factor‐alpha (TNF‐α) inducing HUVEC to produce ROS, resveratrol can activate the SIRT2 pathway to increase the expression of SIRT2, reduce the production of ROS under the above conditions and inhibit the negative effects of oxidative stress.^82^

### 3.2 SIRT2 and oxidative stress‐induced endothelial cell damage

Oxidative stress‐induced endothelial cell damage is inseparable from many diseases. In addition to the aforementioned atherosclerosis, there are also hypertension, peripheral vascular disease,^83^ and diabetes.^80^ The result of oxidative stress occurring in endothelial cells is mainly to promote their own apoptosis and cause the death of endothelial cells.^84^ In the experiment of vascular endothelial injury induced by high glucose, SIRT2 can prevent the injury by inhibiting the p53 and NF‐κB pathways.^85^ But what is interesting is that depending on the degree of oxidative stress, SIRT2 may have two completely opposite effects. Because SIRT2 can bind FOXO3a and make it deacetylated, and FOXO3a will increase the expression of the target protein manganese superoxide dismutase and BCL‐2 interacting mediator of cell death (Bim).^86^ Among them, the former has an antioxidant effect,^87^ which can reduce the level of ROS in the cell, showing a protective effect, while the latter is a pro‐apoptotic protein,^88^ which causes apoptosis and shows a harmful effect. When endothelial cells are stimulated by a lesser degree of oxidative stress, the effect of manganese superoxide dismutase prevails. When the oxidative stress is more intense, Bim's effect will be greater.

### 3.3 SIRT2 and vascular remodelling

In the case of changes in the internal and external environment, in order to ensure the normal supply of blood flow, the blood vessel wall can change its structure to maintain an appropriate lumen size and allow blood to flow smoothly throughout the body. This process is called vascular remodelling.^89^ Vascular remodelling is associated with many diseases or conditions, such as pregnancy,^90^ pulmonary artery blood pressure,^91^ ageing. As mentioned earlier, MTs in cardiomyocytes are related to the occurrence of diabetic cardiomyopathy. There are also MTs in HUVCE, the structure of which is the same as that in cardiac muscle cells. Similarly, when α‐tubulin is deacetylated by SIRT2, the stability of MTs will be greatly reduced, and the migration ability of endothelial cells will be enhanced, which suggests that SIRT2 is a key regulator of vascular remodelling.^92^ In the presence of small trauma in vitro, the enhanced endothelial cell migration ability helps repair skin damage, so SIRT2 plays an active role in this situation.^93^

### 3.4 SIRT2 and energy metabolism

Normal energy metabolism is a prerequisite for maintaining cell physiological functions. Among them, glucose metabolism and lipid metabolism are the most important.^94^ Disorders of energy metabolism of vascular endothelial cells can damage the functions of endothelial cells and cause various vascular diseases.^95^ If this metabolic disorder occurs in the coronary arteries, then some physiological functions of the heart will also be affected.^96^ SIRT2 can inhibit the degradation of phosphoenolpyruvate carboxykinase (PEPCK‐C) to regulate gluconeogenesis.^15^ SIRT2 can also inhibit the acetylation of FOXO1 and promote its combination with PPAR‐γ to inhibit fat formation.^97^ After energy restriction (ER) on Sprague‐Dawley male rats fed a high‐fat diet for 12 months, it was found that the expression of SIRT2 was suppressed. This indicated that there is a link between SIRT2 and energy metabolism.^98^ Experiments have found that the use of SIRT2 inhibitors will cause a significant decrease in the survival rate of porcine vascular endothelial cell line (PIEC). Treatment with SIRT2 inhibitors will increase the apoptosis and necrosis of PIEC, and the content of ATP in the cells will also decrease, indicating that under physiological conditions, SIRT2 is essential to maintain the normal energy metabolism of endothelial cells and maintain their survival of.^99^

## 4 CONCLUSION

**FIGURE 1:**
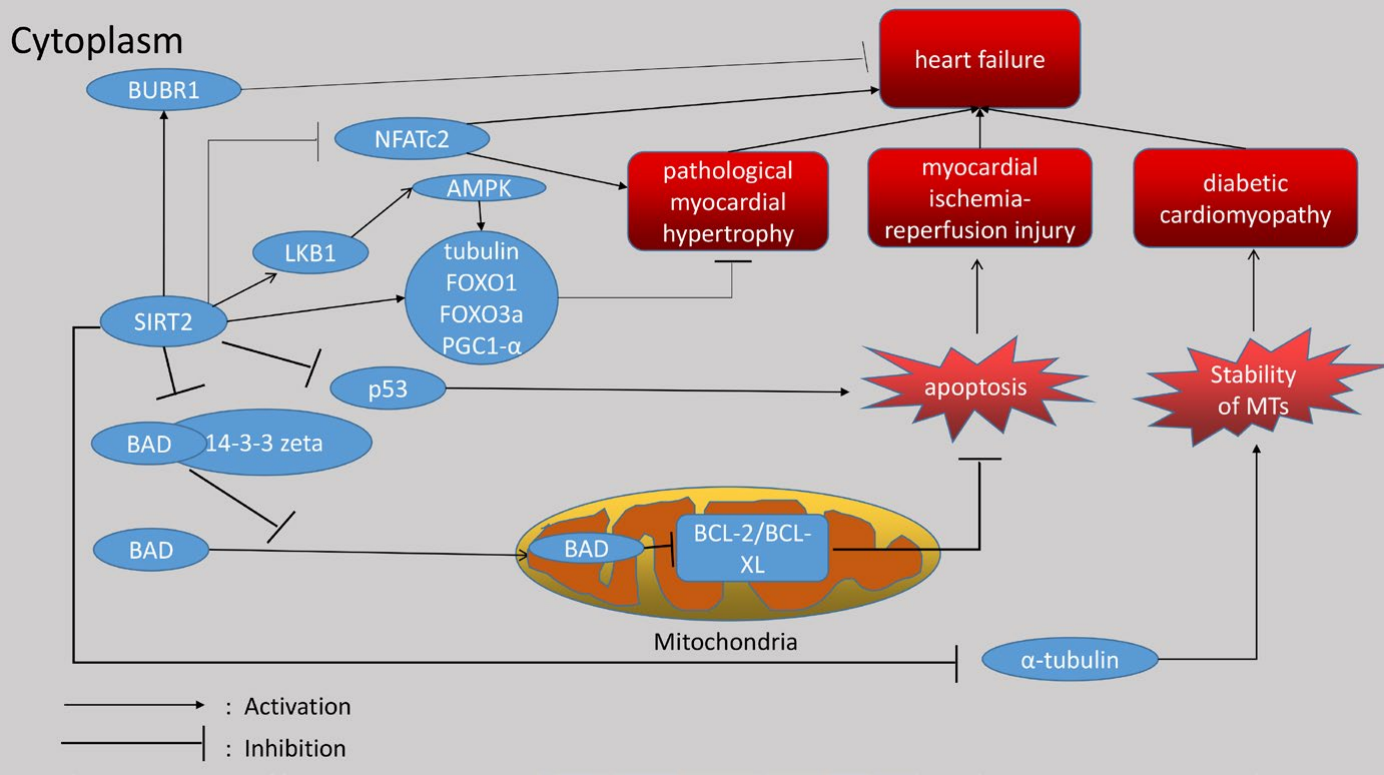
The figure shows that SIRT2 as a deacetylase has a role on a variety of proteins, which ultimately affects the heart in different ways

**FIGURE 2:**
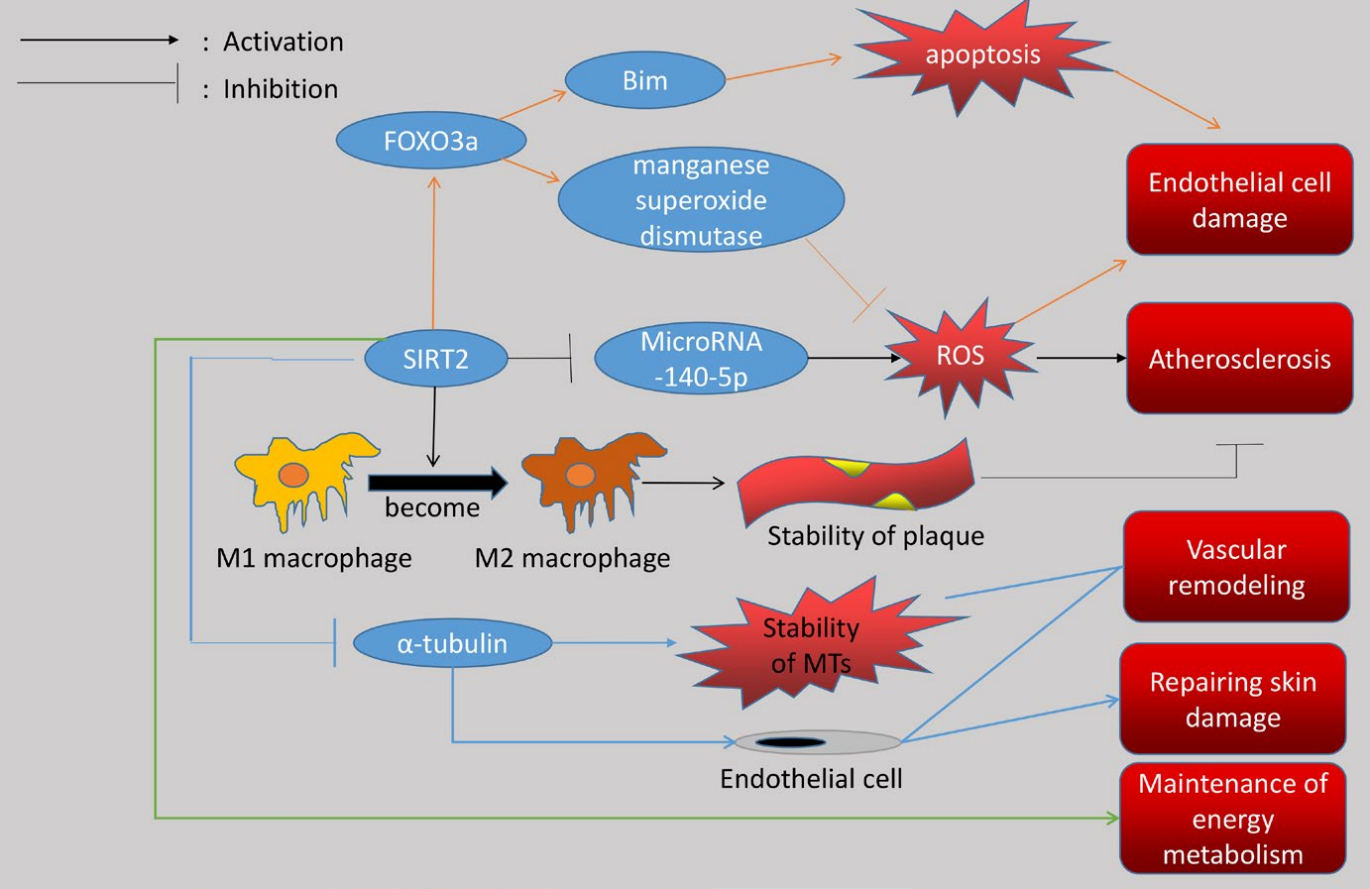
The figure shows that SIRT2 as a deacetylase not only affects a variety of proteins, but also changes the type of macrophages. Interestingly, SIRT2 has two effects after deacetylation of FOXO3a, which is related to the degree of stimulation given to blood vessels. When blood vessels are stimulated by mild oxidative stress, the manganese superoxide dismutase pathway plays a major role, and when blood vessels are stimulated by severe oxidative stress, the Bim pathway plays a major role

In fact, SIRT2 plays a key and important role in cardiovascular diseases, and there are many aspects of the protein available for research, which is very useful for clarifying the future research direction and finding effective clinical drugs for treating cardiovascular diseases. As mentioned above, Table 1 lists the cardiovascular diseases related to SIRT2 and the corresponding molecules that interact with SIRT2. We can see that SIRT2 is involved in many diseases of the cardiovascular system. Figures 1 and 2 vividly introduce the mechanism of SIRT2's participation in cardiovascular diseases. At the same time, it can be seen that SIRT2 has a big difference in the impact of cardiovascular diseases, and sometimes the impact on the same disease is not even the same. This situation has a great relationship with the degree and type of stimuli suffered by the cardiovascular system, and the specific situation requires researchers to do further research. In short, SIRT2 provides a very promising idea for researchers to find drugs for treating cardiovascular diseases.

**TABLE 1:** This table summarizes the effects of SIRT2 on different cardiovascular states and shows the corresponding proteins that are currently known to interact with SIRT2

Disease	Known effects	Related molecule and Ref.
Pathological myocardial hypertrophy	Inhibition	LKB1,^26^ tubulin,^27^ FOXO3a,^9^ FOXO1 and PGC1‐α,^28^ NFATc2,^29^ CSN6,^30^ PHF19^31^
Myocardial ischaemia‐reperfusion injury	Inhibition/Promotion	14‐3‐3 zeta,^38^ BAD,^38^ p53^39^
Diabetic cardiomyopathy	Inhibition	α‐tubulin^45^
Heart failure	Inhibition	BUBR1,^64^ NFATc2^29^
Atherosclerosis	Inhibition	MicroRNA‐140‐5p^81^
Oxidative stress‐induced endothelial cell damage	Inhibition/Promotion (according to the degree of oxidative stress)	p53,^85^ NF‐κB,^85^ FOXO3a^86^
Vascular remodelling	Regulation	α‐tubulin
Energy metabolism in endothelial cells	Maintain normal energy metabolism	PEPCK‐C,^15^ FOXO1^97^

## CONFLICT OF INTEREST

The authors declare that there is no conflict of interest regarding the publication of this paper.

## AUTHOR CONTRIBUTION

**Boquan Wu:** Writing‐original draft (lead); Writing‐review & editing (lead). **Shilong You:** Formal analysis (supporting). **Hao Qian:** Formal analysis (supporting). **Shaojun Wu:** Methodology (supporting). **Saien Lu:** Formal analysis (supporting). **Ying Zhang:** Resources (equal). **Yingxian Sun:** Resources (equal). **Naijin Zhang:** Resources (equal).
